# Comparing and contrasting barriers in augmentative alternative communication use in nonspeaking autism and complex communication needs: multi-stakeholder perspectives

**DOI:** 10.3389/fpsyt.2024.1385947

**Published:** 2024-06-10

**Authors:** Shu H. Yau, Kaylynn Choo, Jane Tan, Olivia Monson, Stephanie Bovell

**Affiliations:** ^1^ School of Psychology, Murdoch University, Perth, WA, Australia; ^2^ School of Education, University of Bristol, Bristol, United Kingdom

**Keywords:** augmentative alternative communication, autism, communication partners, complex communication needs, minimally verbal, nonspeaking, stakeholder perspectives

## Abstract

Augmentative alternative communication (AAC) devices or systems are often prescribed to minimally verbal or nonspeaking autistic individuals and other individuals with complex communication needs to facilitate communication or as an alternative to spoken language. AAC use can result in communication gains and improved quality of life for minimally verbal or nonspeaking individuals. Despite this, AAC abandonment is high, limiting societal participation of the individual on the autism spectrum with complex communication needs. Our study is a novel exploration of the barriers of AAC use from a multi-stakeholder perspective, and a qualitative analysis of similarities and differences between stakeholders. We conducted semi-structured interviews and focus groups with 30 parent-carers, educators and clinicians currently supporting AAC users in Western Australia and analysed the data using reflexive thematic analysis. Barriers from each stakeholder group were coded, resulting in 17 subthemes forming five main themes common to all stakeholders: Stakeholder Knowledge, Stakeholder Attitudes and Stigma, Resources, AAC User Engagement, and Device Fit. Contrasting perspectives included actual and perceived stigma associated with AAC use (parent-carers vs clinicians); different struggles with resources and knowledge (parent-carers vs clinicians and educators); and a lack of clinician communication in the processes that determined AAC-fit for school environments (educators only). Findings are discussed in the context of improving inter-stakeholder collaboration and capacity building in Australian health service and practice to better support minimally verbal or nonspeaking autistic individuals and individuals with complex communication needs. Suggestions are also offered for communication partner training.

## Introduction

Autism spectrum disorder (ASD) is a neurodevelopmental condition characterised by challenges with social communication and heterogeneous language ability (1). About a third of autistic children and youth are minimally verbal or nonspeaking and are often neglected in autism research (2). Minimally verbal or nonspeaking autistic individuals and individuals with significant language needs are encompassed by the broader term complex communication needs (CCN), which affects an estimated 1 in 500 people in Australia (3).

Augmentative and alternative communication (AAC) is typically prescribed as communication interventions for minimally verbal autistic individuals and individuals with CCN (4, 5). AAC encompasses systems and devices that supplement/augment language development or act as a replacement/alternative to verbal speech, or both (6). AAC interventions include unaided (e.g., hand signs) or aided (e.g., speech generating devices) systems, and can range from light-tech (or no-tech) to high-tech systems. The current study focuses on the latter (e.g., iPad, eye-gaze systems), and is aligned with the belief that the goal of AAC is to allow users to communicate independently without a facilitator being present (7, 8).

Research on AAC use shows improved communication skills in autistic children (9) – including those with intellectual disabilities and CCN (10, 11) – decreased challenging behaviours (12, 13), increased requesting skills (14), increased social participation (15), and increased language and communication development (16–19). When interviewed, AAC users and stakeholders reported qualitative benefits like improved communication (20), better parent-child relationships (21), and increased independence (22).

Despite the potential positive outcomes from AAC use, 30%-50% of users abandon or under-use their AACs (23). In a systematic review on barriers and facilitators of light-tech AAC use, Moorcroft et al. (24) identified environmental factors (e.g., attitudes and supports by professionals, family and society) and personal factors (e.g., AAC user’s attitude, socioeconomic status and culture) as the main barriers to provision and use of AAC by people with CCN. Research on barriers of AAC use have found similar themes but often focuses only on AAC users (25), parents only (26–29), or parents and clinicians only (30–32). Our study takes a novel multi-stakeholder approach (parent-carers, educators and clinicians) to capture nuances between stakeholder views on AAC barriers across a wider range of real-life settings (i.e., home, school, clinical therapy).

## Methods

### Participants

This study was approved by the Murdoch University Human Research Ethics Committee. Participants were recruited through purposive sampling through autism-specific services and disability service providers in Western Australia (WA), word-of-mouth, and social media. Participants included nine parent-carers, ten educators and eleven clinicians from metropolitan Perth and regional Western Australia.

Participant demographics can be found in **Table 1**. The parent-carer group comprised primary carers of high-tech AAC users who are predominantly autistic (minimally verbal/nonspeaking) pre-school and school-aged children. The educator group comprised school principals, mainstream and special schoolteachers and assistants. The clinician group comprised speech and language pathologists (SLPs) and psychologists. Educators and clinicians varied in work-experience with AAC users, and types of AAC supports they had engaged with. All clinicians and educators had worked with minimally verbal autistic AAC users of all ages and with varied co-occurring diagnoses. Few had experience with unaided systems (Makaton, AUSLAN), many had experience with light-tech (e.g., PODD) and all had experience with high-tech AAC (e.g., eye gaze, iPad speech generating devices).

**Table 1:** Demographics of 30 participants in focus groups and interviews.

Stakeholder Group	Participant	Role	Child Age (years)	Child Diagnosis	Highest Education Level	AAC experience (years)
Parent-Carer	1	Mother	14	Down syndrome	bachelor	>10
Parent-Carer	2	Mother	14	cerebral palsy	bachelor	NA
Parent-Carer	3	Mother	7	ASD	postgraduate	3-5
Parent-Carer	4	Mother	13	ASD, Bainbridge-Ropers syndrome	bachelor	>5
Parent-Carer	5	Mother	26	ASD	bachelor	>10
Parent-Carer	6	Grandmother	27	ASD	Year 10	>10
Parent-Carer	7	Mother	8	ASD	bachelor	3-5
Parent-Carer	8	Mother	4	ASD	N/A	N/A
Parent-Carer	9	Mother	3.5	Angelman syndromic form of ASD	N/A	N/A
Educator	1	educator	Varies	Varies	postgraduate diploma	>5
Educator	2	educator	Varies	Varies	masters	>10
Educator	3	educator	Varies	Varies	advanced diploma	>5
Educator	4	educator	Varies	Varies	TAFE	3-5
Educator	5	educator	Varies	Varies	graduate diploma	>5
Educator	6	educator	Varies	Varies	bachelor; graduate certificate	NA
Educator	7	educator	Varies	Varies	bachelor; graduate certificate	10-15
Educator	8	educator	Varies	Varies	diploma	10-15
Educator	9	educator	Varies	Varies	PhD	>5
Educator	10	educator	Varies	Varies	diploma	>10
Clinician	1	SLP	Varies	Varies	graduate certificate	5
Clinician	2	SLP	Varies	Varies	postgraduate	>10
Clinician	3	SLP	Varies	Varies	bachelor	<2
Clinician	4	SLP	Varies	Varies	masters	>10
Clinician	5	SLP	Varies	Varies	masters	3-5
Clinician	6	SLP	Varies	Varies	masters	<2
Clinician	7	SLP	Varies	Varies	bachelor	<2
Clinician	8	SLP	Varies	Varies	bachelor	>5
Clinician	9	SLP	Varies	Varies	bachelor	<2
Clinician	10	school psychologist	Varies	Varies	masters	>10
Clinician	11	school psychologist	Varies	Varies	masters	>10

SLP, speech language pathologist; ASD, autism spectrum disorder; ID, intellectual delay.

### Data collection

We used semi-structured interviews and focus groups separated by stakeholder group (i.e., parent-carers, educators, and clinicians) to encourage open conversation in the absence of the other stakeholder groups and therefore a deeper understanding of the challenges unique to each group (e.g., 33). Interviews were targeted to each stakeholder group as per focus group/interview recommendations (34). Participants were asked ten questions in three sections. Section 1 and 2 focused on a stakeholder’s experiences when supporting AAC users. In section 2, participants were also shown nine barriers identified by previous research (21, 24, 35, 36), and asked to rank the three biggest barriers in AAC they had faced (barrier cards are provided in the **Supplementary File**). These rankings had two purposes: first as a conversation tool, inviting participants to agree or disagree on previously found barriers and to elaborate on their points; second as a tool in our analysis to compare and contrast the barriers across the stakeholder groups. Section 3 focused on overcoming barriers which forms a separate study. An assistant moderator was present to record field notes and provide a summary at the end of each focus group or interview. Participants could confirm or correct the accuracy of the summary.

### Procedure

Due to COVID-19 restrictions in WA during 2022, four sessions were in-person and 16 sessions were online. Participants were given the option to attend focus groups (n = 18) or individual interviews (n = 12) as flexibility is needed when collecting data from these populations. This format variability is not uncommon (30, 37) and a breakdown of attendance format by participant group is provided in the **Supplementary File**. To maintain consistency, all researchers piloted sessions with Murdoch University Child Cognition and Autism Research Laboratory members (including those with lived-experience of ASD and/or CCN) who were not involved in the study. Consent was obtained from all participants prior to the study and participants were given a $20 grocery voucher each as a token of appreciation. No participants chose to withdraw post-interview, therefore the final analysis consisted of the full dataset of responses. Participant sessions were recorded and transcribed verbatim.

### Data analysis

Themes were identified as per Braun and Clarke’s (38, 39) reflexive thematic analysis procedures which included researchers’ engagement with semantic content of the data. Using a codebook approach, we first combed the transcripts for potential codes, on MAXQDA 2022 (40). Then, through iterative discussions and inductive data engagement the research team generated and refined themes from the initial codes (41). Finally, we looked for similarities and differences between the stakeholder groups for each of the 18 codes, while also referring to assistant moderator field notes and participants’ top three barrier rankings. Through reflexive discussions, all researchers could debate and challenge different researcher standpoints of the themes (39, 42).

## Results

**Table 2** shows the subthemes generated from all stakeholders that formed the five main themes: Stakeholder Knowledge, Stakeholder Attitudes and Stigma, Resources, AAC User Engagement, and Device Fit. There were differences between stakeholder groups in how each of the five themes were experienced. Subthemes were highlighted if identified as being unique to a particular stakeholder group (italicised in **Table 2**).

**Table 2:** Barriers of AAC use identified by carers, educators and clinicians.

Themes	Codes – Barriers
Stakeholder Knowledge	1. Insufficient access to specialised training (AAC, profound Autism) 2. Lack of awareness or access to disability services. 3. Societal AAC knowledge or awareness 4. *Difficulties in accessing knowledge and technology [parent-carers only].*
Stakeholder Attitudes and Stigma	1. Reluctance in AAC uptake as early intervention 2. Device is withheld for safekeeping *3. Experienced stigma versus perceived stigma [parent-carers and clinicians].*
Resources	1. Limited time 2. Limited financial resources. *3. Time and funds for upskilling and to buy spare devices [educators and clinicians]*
AAC UserEngagement	1. Acute factors 2. Ingrained factors 3. Late AAC introductory age. *4. User characteristics and willingness not a barrier [educators and clinicians].*
Device Fit	1. Poor fit to users due to features 2. Poor fit to context and communication partners. 3. *Poor fit in school due to lack of inter-stakeholder communication [educators only].*

Differences between stakeholder groups are denoted in italics. Differences that arose from or pertained to a specific stakeholder group is noted in square brackets.

### Theme 1: Stakeholder Knowledge

All stakeholders mentioned a lack of AAC knowledge as a barrier. 44% of parent-carers, 90% educators and 55% clinicians ranked this in their top three barriers of AAC use. When stakeholders lacked technical and practical knowledge in AAC, there would be fewer and briefer AAC conversations with users. For example, Parent-Carer 1 said that because she did not know how to operate the AAC device efficiently, it shortens the conversations she has with her child: *“…I try to find the words [on AAC], it takes a long time and [child] loses his patience.”* Lack of knowledge among professionals also leads to poorer support and learning opportunities for AAC users. For example, Educator 5 felt limited in his ability to teach his students who use AAC if he is not fluent in using the device *“How are you supposed to model it and make it useful and valuable to the students?”.*

All stakeholder groups unanimously raised that their lack of knowledge was attributable to difficulty accessing training. Some parent-carers faced hurdles at the beginning of their AAC journey, as they could not readily access AAC or speech and language services and relevant training, *“We were very new to NDIS [National Disability Insurance Scheme] and we didn’t know where we were meant to get any of the services from.”* (Parent-Carer 6). Many clinicians cited a lack of depth in AAC training during tertiary education made them feel poorly prepared to serve AAC users and their families. *“[There are] theoretical things in university, but there’s not much opportunity to apply in practice the actual selection of device, selection of vocabulary, display.”* (Clinician 3). This sentiment is echoed by Clinician 1, “*(at university) we never touched a device … never done a disability practical…”.*

A lack of AAC knowledge in stakeholders in our study also extended to other communication partners such as peers, siblings and the broader society. Parent-Carer 5’s son communicates with his parents, support worker and SLP using AAC, however, his AAC interactions are limited when he is within community settings (e.g., work and peer-support groups) *“…takes time for him to get [AAC] out and start it up. If they don’t know he’s using it, it can end the conversation a bit quickly.”* The same occurs in therapy,*“…a lot of talking by the occupational therapist and [name]’s got less opportunity to talk because he’s not using the device … unless the support worker specifically intervenes and suggests the use of the iPad, it’s not used at all.”* (Parent-Carer 5).

Additionally, psychologists in our clinician sample questioned their role in the AAC space, “*Is it to advocate for its use? Is it to incorporate its use in what we do? This is a speech space and we’re a bit turf aware…(knowing the boundary) can help us be more respectful of our clients and more consistent with APS (Australian Psychological Society) ethics … the ethical code has things on disrespectful communication and respect, and I think AAC is part of respectful communication*.” (Clinician 10).

#### Stakeholder Knowledge: parent-carer specific barrier

Four parent-carers (44%) described facing additional challenges, such as understanding AAC training, autism research and in navigating high-tech AAC devices: “*I’m not terribly well-educated. But (AAC trainers) presume that everyone’s got a university degree … a lot of us … left school in year ten, and we really aren’t up with all this modern stuff. They assume you know a lot about the current research on autism…”* (Parent-Carer 6).

### Theme 2: Stakeholder Attitudes and Stigma

88% of parent-carers, 80% of educators and all clinicians mentioned experiencing negative attitudes towards AAC use and uptake. Stakeholders raised concerns that AAC would hamper a child’s development and potential, and more pragmatic concerns around being responsible for damaging expensive equipment. For example, parent-carers cited their own initial hesitation when AAC was suggested as an intervention: “*I worried my son will lose his will to speak, so better to make him speak clearly, use more speech therapy.”* (Parent-Carer 1). However, parents in our sample eventually jumped onboard with AAC intervention when they experience success communicating with their child, “*I wasn’t ready for it (AAC), seems confronting (to think) oh she’ll never speak, but she took to it well and it really let us see that she is thinking about things and has things to tell us. She just can’t say that verbally … it’s given her a voice.”* (Parent-Carer 2).

Parents cited further hesitation to AAC intervention when clinicians they rely on for advice held beliefs that a young child was not ‘capable’ or ready for AAC: “*The paediatrician (said) you can’t give her [child] a communication system, she won’t understand it. She needs to first show that she can understand pictures.”* (Parent-Carer 8). Clinicians noticed that this then causes delays to communication intervention, “*Often paediatricians won’t refer for an assessment for AAC and will try manage what I would call complex and challenging behaviours through medications.*” (Clinician 11).

Lastly, all stakeholder groups experienced communication partners withholding the device, due to a fear the device would ‘break on their watch’, “…*mum that doesn’t let her daughter use her device outdoors because she’s afraid it will get broken … fear of that ‘gap’ when it’s broken, and you have to get it replaced or fixed.”* (Clinician 5). Parent-carer 3 states, *“…a few times our therapist mentions that A’s tablet is not easily accessible for him at school, like it’s up in the cupboard … the school was concerned the tablet got damaged, not necessarily by A, but you know, because there’s other kids*”.

#### Stakeholder Attitudes and Stigma: experienced stigma versus perceived stigma

Another concern that was evident across the dataset was the stigma associated with being seen to use an AAC device. Parent-carers were worried about negative societal perceptions or societal stigma against their children using AACs. *“…(people) don’t expect him to have a device, or don’t understand why he doesn’t talk straight away because he looks like everyone else…”* (Parent-Carer 6). Some parent-carers experienced barriers in communication from extended family members and peers because of the AAC, *“…cousins don’t try at all to communicate with (child), and kids in general don’t, they just sort of go, ‘oh she can’t talk’”* (Parent-Carer 4).

In contrast, clinicians believed this to be an issue of ‘perceived stigma’,*“I don’t think it’s society not accepting it. I think it’s parents thinking society won’t accept it.”* (Clinician 1). Clinicians mentioned stories of AACs going unused due to stigma that carrying an AAC signals that one is ‘incapable’, *“For years … nobody would ever set up his device for him because they [believed] … makes him look more disabled or makes him look more different [in school].”* (Clinician 1). Clinicians also reported having seen acceptance and patience: *“A lot of adults generally are accepting, they would wait … and would give [AAC users] the time to say what he needs”* (Clinician 5). This was also reported in children, *“Their peers absolutely love it (speech generating AAC) … other kids are like, what have you got and can I press it, can I play too?”* (Clinician 4).

As Clinician 10 surmises, stigma may be due to a lack of visibility of high profile AAC users in Australia, *“We have Dylan Alcott, but we don’t really have a champion for AAC”.*

### Theme 3: Resources

All stakeholders cited competing financial and time demands that limit the quantity and quality of AAC opportunities they could create. Parent-carers experienced competing financial and time demands, and need to prioritise care-related needs over AAC communication: “*…her needs are very large so we don’t have enough money to have a speechie and also have a physio, ABA … I haven’t got the time or the resources to create little booklets and read with her with her device … everything takes a very long time, plus working and my other child … you’re so busy trying to feed her, toilet her, get her to sleep…”* (Parent-Carer 4).

Educators and clinicians also cite a lack of resources as a barrier, specifically in family members and support workers. “*Support workers are paid to prioritize housework over working with people on their communication.”* (Clinician 8). Clinician 3 adds, “*(families) have so much going on in their lives, so many stressors than learning a new language system … they’re sleep poor, time poor. It’s hard to be adding more.*”

While educators and clinicians do not think financial resources are a barrier for them per se, most raised that organisational decisions ultimately affect whether they can attend AAC training or have resources to work with: *“It’s really hard to get to know [AAC system names] when you’re in class with 30 kids … already under the pump to get your curriculum boxes ticked, let alone stop and try to learn a device … when that child goes, the device goes. When do you ever practice? Do you take it off a child during recess and practice? No, I need spare devices.”* (Educator 4).

### Theme 4: AAC User Engagement

In our coding, most stakeholders (89% of parent-carers, 60% of educators and 55% of clinicians) identified a user’s willingness to use the AAC as a barrier. *“(Name) has the ability and knows where most of the words are. But his willingness to engage … Or wanting to use it as a communication. That’s the biggest barrier.”* (Parent-Carer 7).

When an AAC user does not initiate or reciprocate communication using AAC, it can be due to either acute or ingrained reasons. An acute example is when a child is emotionally dysregulated: *“If he is sad or angry … he doesn’t have the concentration to look at the AAC … the time that you really want to communicate to find out what is wrong is the time that we have the most difficulty in in trying to reach him*” (Parent-Carer 3). Autistic AAC users may rely on ingrained or internalised routines, and not spontaneously initiate AAC use: “… *their routine of using their device is, someone tells me to press the button and then I press it … they internalize that as how they use their device … prompt dependent …”* (Clinician 4). Other autistic children may use AAC only in specific or predictable contexts: *“… when I use the AAC with him (to chat), he just doesn’t want to use it. If I said let’s do your homework … then he will use his AAC.”* (Parent-Carer 7). User engagement in our sample was compounded by the age at which the AAC device was introduced: “*When devices are introduced late, they [users] have already established pretty effective means of communicating their needs and wants.”* (Educator 5).

#### AAC User Engagement: more of a barrier to parent-carers than educators and clinicians

When asked to rate the biggest three barriers that affected their AAC use, nine parent-carers (100%) picked ‘user engagement/willingness’ compared to educators (30%) and clinicians (27%). When asked to elaborate, educators and clinicians believed that user (dis)engagement is ‘perceived’ and the real barrier is poor device fit and support.

*“…individual’s abilities or willingness should not be up there [of top barriers]. It might look like that, but it’s because they’ve been given the wrong system or the people around them haven’t got adequate training to support its use.”* (Educator 6).

### Theme 5: Device Fit and features

‘Device Fit’ was rated as a top three barrier to AAC use by all parent-carers, 80% of eight educators and 73% clinicians, due to poorly customised fit or sensory overwhelm to the user.

*“Ideally we’ll have the AAC on him all the time, like having the tablet sling on his shoulder … but it does hinder his movement.”* (Parent-Carer 3 on the bulkiness of the AAC).

*“There were just too many (distracting) icons”* (Educator 8 on why they abandoned a high-tech AAC for an autistic child).

*“… the voice … is robotic or American (in an Australian context)… not representative voice” (*Educator 10*)*

Other times, the device or AAC system prescribed for a child is not the ‘supported AAC type’ in their school, which hinders their daily use *“… it just happened that LAMP [prescribed AAC] is not the preferred system, [school] prefers Proloquo … but it’s not like we can swap our system, the time and energy costs of switching systems is not worth it”* (Parent-Carer 3)

#### Device Fit educator specific barrier of poor inter-stakeholder communication

While all groups discussed poor device fit to the child’s daily use, only the educator group brought up that this was due to a lack of inter-stakeholder communication. Educators mentioned that schools are generally not involved or consulted when a child’s device is being chosen by clinicians, resulting in a device presenting barriers at school, where a child spends a significant amount of time, *“The devices would just turn up … (we all agree) that is not an appropriate choice for that person (in school).”* (Educator 5).

## Discussion

In this study, we set out to identify and contrast barriers to AAC use in a multi-stakeholder group (parent-carers, educators and clinicians) who support an autistic child who is nonspeaking or minimally verbal, as well as those with CCN.

The first theme on poor *Stakeholder Knowledge* was consistent with findings in parents (29), educators (36) and clinicians (43). In a reversal of findings to ours, ‘lack of knowledge’ was brought up in 80% of caregivers but only 40% of clinicians in Romano and Chun's (31) study. This difference is likely due to work experience in our sample, with many of our educators and clinicians having two or less years of AAC experience whereas Romano and Chun sampled ‘experienced’ speech pathologists. This reflects the role of work experience in increasing confidence and efficacy in clinicians and suggests the importance of pre-service (44) and in-service training. Higher knowledge and self-efficacy in clinicians are often related to more experience working with autistic clients and specific training in autism and complex co-occurring conditions (45). While all our stakeholders cite difficulties accessing specialized training and disability services, our parent-carers mentioned additional challenges in understanding the content within AAC training and navigating high-tech AAC devices. Ganz et al. (46) suggested that service provision and AAC selection need to consider the individual with CCN, as well as the preferences of the key stakeholders that support them. In this case, it is imperative for clinicians to take extra time to identify parents’ level of knowledge and consider the technology they are comfortable with, when prescribing AAC and designing training for them.

Our second theme was on *Stakeholder Attitudes and Stigma.* As with past studies (4, 9, 13, 16, 20, 22, 24, 27, 34, 35, 46–52), our parent-carers initially worried that their children will lose their potential for speech if they rely on AAC devices to ‘speak for them’, or that AAC devices will single their child out to peers as being different. The former is linked to stakeholder attitudes (i.e., belief in myths; 53), and the latter to stigma. Within our second theme, there was a sharp contrast in the experience of societal stigma: parent-carers acknowledged a real impact, whereas clinicians attributed it to perception.

It is possible that the discrepancy between ‘real’ and ‘perceived’ societal stigma is largely context-dependent. In the broader community (parent-carer context), there is limited knowledge of and exposure to AACs and people with disabilities, in comparison to structured school programs or speech-language clinics (educator and clinician context). The communities within the latter context are inherently more inclusive due to relevant professional training and/or exposure to disability, making it less likely for educators and clinicians to encounter the stigma and isolation that parent-carers experience: an area that warrants future research. Future research should also investigate the potential influence of the AAC user’s age or AAC device type on the different experiences of stigma. For instance, clinicians who work with younger children or children with mainstream devices (e.g., ipads) may have more positive experiences. Societal stigma may be reduced by improving AAC interventions through peer-mediated interventions with explicit teaching of AAC use and turn-taking in children (49, 54). When neurotypical peers were taught AAC interaction strategies, students using AAC enjoyed their interactions, saw their peers as friends, and were more involved in class activities (55).

Also concerning is that our parent-carers and clinicians were discouraged from requesting AAC intervention for children by physicians who cited inaccurate attitudes and beliefs that children needed ‘pre-requisite’ cognitive and sensorimotor skills to use AAC. This is likely linked to physicians’ self-reported lack of autism-specific knowledge and confidence in managing care of autistic individuals with co-occurring intellectual delays or other severe impairments (51, 56). This calls for targeted development of autism and complex communication training programs focusing on improving physician awareness, efficacy and behaviours. Lastly, there were pragmatic concerns around being responsible for expensive AAC equipment, and being fearful of being penalized or going without, in the event the AAC is damaged or lost. This suggests a need for clarity in NDIS provisions [e.g., device replacements; (57)] and is linked to the next theme on resources.

Our third theme on *Resources* (a lack of or competing) was consistent throughout parent-carer, educator and clinician groups. Our parent-carer group mentioned competing demands on their time and finances, making it hard for them to commit to AAC partner training. Indeed, it is not uncommon to find parents in AAC families performing the roles of caregivers, communication partners, teachers, advocates, therapy coordinators and AAC programmers (58). Our educators and clinicians were also affected by a resource constraints, such that the time available for training families and communication partners is dependent on a family’s funding (NDIS or otherwise). Moreover, their own time and ability to access resources to upskill and practice on devices are tied to organizational decisions.

The fourth theme on *User Engagement* describes barriers to effective AAC use when a child is dysregulated or prompt-dependent – which was also found by Donato et al. (47). Parent-carers in our study who find that their autistic children have a prescribed use of AAC is consistent with research in families with minimally verbal children with autism, where AAC use is primarily transactional (e.g., food/drink requests; 33). Unexpectedly, our educators and clinicians did not consider user engagement as a barrier to AAC use, which contrasts with previous findings (e.g., 43, 59, 60), as well as our parent-carer experiences. This could be explained by our earlier finding on autistic children’s lower engagement in social communication versus their preference or better performance in task-oriented communication and context-bound routines. Educators and clinicians often interact with autistic children within a structured program with goal-oriented tasks, which autistic children typically perform better at/engage in more (61), hence professionals in our study may not experience the user (dis)engagement that parent-carers do.

The fifth and final theme is on *Device Fit* for the user, specifically physical or sensory mismatch, also found in other studies (28, 59). A few parents and clinicians mentioned AAC users eschewing their speech generating devices due to the identity or pitch of the voice. Promisingly, AAC technology is developing, where ‘voices’ can be customized using vocalizations from the user combined with recordings of a matched-speech donor (62). To prevent a family giving up on AAC altogether due to poor fit, it is important that clinicians and service providers communicate clearly with parents and educators that ‘finding the right fit’ is often an ongoing process (50), and encourage them to be flexible and open-minded when trialling AAC systems. Legislation on AAC access and service providers should ideally support changing AAC needs as circumstances and skills inevitably change. This may mean an AAC user needs two different types of AAC concurrently, so they have the freedom to swap to the communication method that works for them in the moment (63).

To be used effectively, AACs also need to fit the main contexts where they will be used daily, such as school. However, our parent-carers reported their child getting less support in school if they had a ‘less supported’ AAC system. Related to this, educators lamented the lack of collaboration and communication when clinicians decide on AAC fit. Such barriers can be eased by implementing an interprofessional collaboration (IPC) framework (64), which is both patient-centred and population‐oriented. In IPC, problem solving is shared at the community level to ensure appropriate access and fit to services for autistic individuals. This may demand more time from educators and clinicians – and could be constrained by limited knowledge and professional boundaries – but is deserving of additional time from employers and funding through government bodies. Implementing ICP can ease the burden of care coordination for caregivers by eliminating care silos. Clinicians who engage in shared decision-making with schools are more knowledgeable about feasible interventions within the constraints of a school setting (52), which better supports the child with the AAC.

The comparative approach of our study highlighted common experiences across the three stakeholder groups as well as contrasting perspectives. These views can be used in future training with the different stakeholders to help break down boundaries and foster the connections needed to improve inter-stakeholder collaboration. It is important to include AAC users themselves in future studies, to further understand their views on barriers in relation to their communication partners. It could also be useful to extend on our findings through micro-ethnography of AAC users with neurotypical peers. Future researchers should also aim to recruit the voices of fathers, support workers and other therapists who are also frequent communication partners.

## Conclusion

While AAC use is beneficial in fostering communication in minimally verbal and nonspeaking individuals, up to 50% of users and families abandon or underuse their AAC. Our study explored barriers to AAC use in different stakeholder groups and found that barriers fell into five themes: *Stakeholder Knowledge*, *Stakeholder Attitudes and Stigma, Resources, User Engagement, and Device Fit.* By employing a multi-stakeholder approach, we uncovered nuanced differences between stakeholders in supporting autistic AAC users and those with complex communication needs. Such insights are useful in tailoring training to meet each stakeholder group’s needs to better support an AAC user. Our findings are important for ongoing Australian NDIS legislative amendments, specifically to improve access to resources and training, and inter-agency collaboration.

## Data availability statement

The raw data is not available due to the video format of the recordings, and potentially identifiable information shared by stakeholders, particularly information from parent-carers on children. However, de-identified transcriptions or data codes supporting the conclusions of this article will be made available by the authors, without undue reservation.

## Ethics statement

The studies involving humans were approved by Murdoch University Human Research Ethics Committee. The studies were conducted in accordance with the local legislation and institutional requirements. The participants provided their written informed consent to participate in this study. Written informed consent was obtained from the individual(s), and minor(s)’ legal guardian/next of kin, for the publication of de-identifiable data included in this article.

## Author contributions

SY: Conceptualization, Data curation, Formal analysis, Funding acquisition, Investigation, Methodology, Project administration, Resources, Software, Supervision, Validation, Visualization, Writing – original draft, Writing – review & editing. KC: Data curation, Formal analysis, Project administration, Writing – original draft, Writing – review & editing. JT: Data curation, Formal analysis, Investigation, Project administration, Resources, Writing – review & editing. OM: Conceptualization, Methodology, Validation, Writing – review & editing. SB: Data curation, Formal analysis, Investigation, Project administration, Resources, Writing – review & editing.
