# Supplementary material for: Comparing and contrasting barriers in augmentative alternative communication use in nonspeaking autism and complex communication needs: multi-stakeholder perspectives
Source: Front Psychiatry. 2024 Jun 10;15:1385947. doi: 10.3389/fpsyt.2024.1385947 (PMC11197385; doi:10.3389/fpsyt.2024.1385947)
Supplement: Supplementary file 1 [file Presentation_1.pdf]

Appendix

Cards distributed or shown on a screen to parent-carers to ask them to rank the top three barriers to their child's AAC use and participation. Themes from Moorcroft et al. (2019a) and Park (2020).

AAC user's abilities/willingness	Support from therapist/clinician/ teacher/professional	AAC knowledge of therapist/clinician/ teacher/professional
Personal and family attitudes on AAC use	Competing demands (time, money, etc.)	Features of AAC systems
Support network (family and friends)	AAC-related service/information accessibility	Fit of AAC system

Cards distributed or shown on a screen to clinicians and educators to ask them to rank the top three barriers to their child's AAC use and participation. Themes from Moorcroft et al. (2019b), Chung and Stoner (2016), and Johnson et al. (2006*)*.

Training/knowledge of AAC	Individual's abilities/willingness	Features of AAC systems
Financial resources	Carers' attitudes on AAC	Fit of AAC system to individual
Support from team/staff	Other carer-related factors (resource, knowledge, support)	Societal acceptance of AAC use

The table below shows a breakdown of attendance format by participant group.

**Focus Groups**	**18**
Clinician	9
Educator	7
Parent	2
**Interview**	**12**
Clinician	3
Educator	2
Parent	7
**Grand Total**	**30**

References

Chung, Y. C., & Stoner, J. B. (2016). A meta-synthesis of team members' voices: What we need and what we do to support students who use AAC. *Augmentative and Alternative Communication*, *32*(3), 175-186. https://doi.org/10.1080/07434618.2016.1213766

Johnson, J. M., Inglebret, E., Jones, C., & Ray, J. (2006). Perspectives of speech language pathologists regarding success versus abandonment of AAC. *Augmentative and Alternative Communication*, 22(2), 85–99. https://doi.org/10.1080/07434610500483588

Moorcroft, A., Scarinci, N., & Meyer, C. (2019a). A systematic review of the barriers and facilitators to the provision and use of low-tech and unaided AAC systems for people with complex communication needs and their families. *Disability and Rehabilitation: Assistive Technology*, *14*(7), 710-731. https://doi.org/10.1080/17483107.2018.1499135

Moorcroft, A., Scarinci, N., & Meyer, C. (2019b). Speech pathologist perspectives on the acceptance versus rejection or abandonment of AAC systems for children with complex communication needs. *Augmentative and Alternative Communication*, *35*(3), 193-204. https://doi.org/10.1080/07434618.2019.1609577

Park, H. (2020). Parents' Experiences and Acceptance Factors of AAC Intervention for Children with Complex Communication Needs. *Communication Sciences & Disorders*, 25(2), 318–333. https://doi.org/10.12963/csd.20729
